# Cell-Friendly Chitosan-Xanthan Gum Membranes Incorporating Hydroxyapatite Designed for Periodontal Tissue Regeneration

**DOI:** 10.3390/pharmaceutics15020705

**Published:** 2023-02-20

**Authors:** Rafael Maza Barbosa, Daniel Navarro da Rocha, Renata Francielle Bombaldi de Souza, Jheison Lopes Santos, José Ricardo M. Ferreira, Ângela Maria Moraes

**Affiliations:** 1 Department of Engineering of Materials and Bioprocesses, School of Chemical Engineering, University of Campinas, Campinas 13083-852, SP, Brazil; 2 Department of Bioengineering, R-Crio Criogenia S.A., Campinas 13098-324, SP, Brazil; 3 Department of Physics, Federal Rural University of Rio de Janeiro, Rio de Janeiro 23890-000, RJ, Brazil

**Keywords:** chitosan, xanthan gum, hydroxyapatite, membranes, mesenchymal stem cells, periodontitis, drug carrier

## Abstract

In this work, a simple method was proposed to produce dense composite polysaccharide-based membranes to be used for guided tissue and guided bone regeneration. The mucoadhesive polysaccharides chitosan (C) and xanthan gum (X) were used to produce polyelectrolyte-based complex membranes. Hydroxyapatite (HA) was added to the formulation as a potential drug carrier, in C:X:HA mass proportions equal to 1:1:0.4, 1:1:2, and 1:1:10, and also to improve membranes bioactivity and biomimetic properties. FTIR analysis indicated successful incorporation of HA in the membranes and XRD analysis showed that no changes in the HA crystalline structure were observed after incorporation. The residual mass evaluated by TGA was higher for the formulation produced at the proportion 1:1:10. The membranes produced showed asymmetrical surfaces, with distinct roughness. Increasing the HA concentration increased the surface roughness. Greater in vitro proliferation of dental pulp mesenchymal stem cells was observed on the surface of the membrane with 1:1:10 C:X:HA proportion. However, the 1:1:2 formulation showed the most adequate balance of mechanical and biological properties. These results suggest that adding HA to the membranes can influence mechanical parameters as well as cell adhesion and proliferation, supporting the potential application of these materials in regenerative techniques and the treatment of periodontal lesions.

## 1. Introduction

Periodontal diseases are related to inflammatory conditions that affect tissues surrounding and supporting the teeth. These periodontium disorders affect 20 to 50% of the population worldwide and may be associated with systemic conditions such as diabetes and cardiovascular disorders, representing a major public health concern [1]. This inflammatory condition can cause a change in the microbial flora present in the oral cavity and favor colonization by periodontopathogens. Colonization by these pathogens further increases the body’s immune response. The presence of cytokines involved in the process of bone regulation and maintenance and the activation of macrophages can, at an early stage, increase the cascade of inflammatory factors for phagocytosis of tissues adjacent to the tooth [2,3,4]. Therefore, microbial control and immunomodulation at the beginning of the disease are essential to prevent the disease from aggravating [3,4]. The treatment of these conditions may vary according to the disease stage or type of tissue affected. In general, at early stages, when the alveolar bone is partially affected, or even in case of severe damage, biomaterials have been used together with regenerative techniques to prevent local and systemic complications. These techniques include guided bone regeneration (GBR) and guided tissue regeneration (GTR), both surgical procedures that combine different types of biomaterials, such as bone grafts and synthetic membranes. These biomaterials act as local bone substitutes or biological barriers intended to direct bone, epithelial, and connective tissue growth to restore the affected tissues and their functions, as well as aesthetic aspects [5,6]. Thin membranes made of collagen, gelatin, and polycaprolactone or their combination with bone grafts are among the most effective biomaterials for GTR, acting as a biological barrier and preventing the formation of fibrotic or scar tissue [7,8,9]. In addition to these combinations of materials and GTR techniques, several bioactive agents can be loaded into the biomaterial to prevent bacterial colonization and growth (e.g., doxycycline), immunomodulatory products (such as medicines or proteins), and stem cells [1,7].

Depending on their biosorption half-life, several types of membranes are used in GBR and GTR techniques, which are mainly categorized as resorbable or non-resorbable materials. Non-resorbable materials, such as membranes produced with expanded polytetrafluoroethylene (e-PTFE), are biocompatible and act as excellent barriers against connective tissue penetration to guide bone tissue regeneration [6,8]. However, a second surgical procedure is required to remove these rigid membranes, which can result in soft tissue dehiscence and susceptibility to infections. In turn, when bioabsorbable membranes produced from natural or synthetic biodegradable materials are used, a second surgical intervention may not be required. In this case, the main clinical and biological challenge is to match the biomaterial resorption rate with neotissue formation [8].

Many efforts have been made toward the development of membranes with tunable degradation rates and adequate biological responses by combining different materials, such as polymers and ceramics, to obtain blends, composites, or matrices with distinct degrees of crosslinking [8]. Examples of biomaterials used for the treatment of periodontal lesions include natural polymers, such as xenogeneic-derived collagen; synthetic polymers, such as poly (lactic acid), and ceramic materials, such as calcium phosphates, e.g., hydroxyapatite and tricalcium phosphates (TCP) [10,11].

The combination of polymers with different properties can generate formulations with improved characteristics. Mucoadhesive formulations, for instance, can be successfully prepared using several types of biodegradable and biocompatible polysaccharides, such as alginate, chitosan, carboxymethylated chitosan, xanthan gum, and alginate [12,13], among others.

The polysaccharides C and X can be combined to produce stable, non-cytotoxic membranes, with enhanced mechanical and physicochemical properties when compared to biomaterials produced with the isolated polymers, being able to be used as scaffolds for cell growth in different tissue engineering applications [14,15,16]. Both polymers have important characteristics when applied in bone tissue regeneration and periodontium-related diseases. X is a biocompatible polymer and is not cytotoxic It shows mucoadhesive capacity in the oral cavity, being up to 3.6 times more mucoadhesive than chitosan [17]. Due to its high aqueous retention capacity, X can be a relevant component of formulations in the form of gels with the application as drug delivery systems for the treatment of diseases that affect the oral cavity, particularly for periodontitis [18]. Chitosan is another non-cytotoxic natural polymer widely used in tissue engineering and combined with HA for bone tissue regeneration [19]. Its antimicrobial properties and capacity to prevent the formation of microbial biofilms, which are related to the etiology of periodontitis, consist of advantageous properties for this particular application [20,21]. At adequate pH conditions, when these polymers are combined, electrostatic interactions occur between chitosan amino groups and carboxyl groups from xantham gum, leading to the formation of a stable polyelectrolyte complex (PEC) that joins the positive properties of both compounds. The PEC may be further stabilized by hydrogen bonds and can be processed as mucoadhesive films, membranes, and sponges. These biomaterials are capable of absorbing high quantities of biological aqueous fluids and culture medium and are able to effectively sustain cells inoculated in them. Thus, chitosan-xanthan gum-based PECs may be considered attractive materials for application in GBR or GTR [22].

Ideally, GBR membranes should not only act as barriers but also show bioactive properties to lead to a specific cellular response and accelerate bone regeneration in the bone defect site. Therefore, the incorporation of calcium phosphates in these biomaterials may consist of a relevant strategy to further improve their mechanical and biological properties for bone tissue regeneration application [22,23]. Hydroxyapatite is a bioactive ceramic compound and, due to its similarity to the bone mineral phase, it has been applied as a bone graft component or as coatings of inert metallic implants [24,25,26]. HA properties, such as biocompatibility and osteoconductivity, can increase cell activity, promoting local bone defect mineralization and repair [22,27]. Some efforts have already been made to produce biomaterials using natural polymers in combination with calcium phosphate. However, these methods may be complex and require different equipment for manufacturing the biomaterial [27,28]. The combination of C, X, and HA may be stabilized by several different interactions in addition to those related to the electrostatic C-X PEC formation, also including interactions between chitosan and HA, involving positively charged amino groups from C and PO_4_^3−^ from HA, xanthan gum carboxyl groups and calcium ions from HA, as well as hydrogen bonding between different groups. Therefore, these interactions may occur in a synergistic way [27], resulting in very stable bioactive biomaterials.

Therefore, the individual and joined properties of each of the compounds explored in this work, mainly those related to periodontal diseases and its repair and regeneration processes, can provide a single biomaterial with ideal characteristics for the regeneration of bone and adjacent tissues. In the present work, chitosan-xanthan gum membrane formulations were combined with HA through a simple method to improve their bioactivity and biomimetic properties. The influence of different concentrations of HA on the morphological, physicochemical, and mechanical properties of the membranes was investigated. In addition, the analysis of the proliferation of dental pulp stem cells (DPSC) seeded on the distinct surfaces of these membranes was evaluated to determine the suitability of the biomaterial for the application as GBR and GTR membranes.

## 2. Materials and Methods

### 2.1. Materials

Chitosan with a deacetylation degree of 83% (product reference 448877, Sigma-Aldrich, St. Louis, MO, USA), glacial acetic acid (product reference 100631000, Merck, Rahway, NJ, USA), and xanthan gum from *Xanthomonas campestris* (product reference G1253, Sigma-Aldrich, St. Louis, MO, USA) were used for the production of the membranes. Lactic acid (product reference 01A1040.01.BJ, Synth, San Francisco, CA, USA), calcium hydroxide (product reference102047, Merck, Rahway, NJ, USA), phosphoric acid 85% (product reference 100552, Merck, Rahway, NJ, USA), and potassium hydroxide (product reference 01H2002.01.AG, Synth, San Francisco, CA, USA) were used for the synthesis of hydroxyapatite. Ultrapure water (Milli-Q Direct Q 8/16 System) was used for the preparation of the solutions. The DPSC (product reference PT-5025, Lonza, Walkersville, MD, USA) were provided by the company R-Crio (Campinas, SP, Brazil).

### 2.2. Production of C-X Membranes Containing HA

The methodology for the fabrication of the bioactive membranes containing HA was divided into 2 steps. First, the production of a solution rich in calcium and phosphate ions by the addition of precursor salts under controlled temperature and pH was performed, as previously reported [24,25,26], and then, the pH was adjusted to 12 to precipitate the HA. In the second step, different proportions of HA were added to the polysaccharide mixture produced under controlled conditions, followed by membrane drying, neutralization, and sterilization. These steps are detailed below.

#### 2.2.1. Synthesis and Processing of Hydroxyapatite

The methodology used to obtain the HA powder was based on the precipitation of the compound in an aqueous medium. The synthesis of HA was carried out in a 1.5 L glass chemical reactor equipped with a mechanical stirrer and systems for pH and temperature monitoring (Tecnal, Piracicaba, SP, Brazil, model Tec-Bio-Flex), under continuous stirring at 300 rpm First, 250 mL of lactic acid (1.1 mol/L) was mixed with 250 mL of calcium hydroxide solution (0.52 mol/L) at a flow rate of 50 mL/min. Then, 250 mL of 0.3 mol/L phosphoric acid solution was added to the previous solution at a flow rate of 8 mL/min. The final solution was heated up to 60 °C in the reactor and the pH was corrected to 12 with 1.4 mol/L potassium hydroxide solution. Finally, the volume was set to 1.0 L with ultrapure water and these conditions were kept constant for 3 h.

After the reaction period, the liquid was filtered through a 3 µm pore size filter paper (Qualy, Prolab, São Paulo, SP, Brazil), under 400 mbar vacuum (DVP, model ZA 60S). The retained precipitate was washed with ultrapure water to remove potassium ions until the pH of the filtered liquid reached 7. The powder was then frozen at −30 °C for 24 h in a freezer (Analytical, model HOTA 20FL) and, later, it was lyophilized at −30 °C (Christ, model Alpha 1-2) for 48 h under vacuum conditions equal to or below 4 mbar. Finally, the powder was grounded using an agate mortar and pestle set and sieved through a 150 µm pore size stainless steel sieve (Bronzinox, São Paulo, SP, Brazil).

#### 2.2.2. Production of C-X Membranes and C-X-HA

The methodology used for the fabrication of C-X membranes was based on previously published works [14,15,16], using a C to X mass ratio of 1:1. Briefly, 100 mL of C solution (at 1% *w*/*v* in acetic acid 1% *v*/*v*) was added to 100 mL of X aqueous solution (also at 1% *w*/*v*) at a rate of 5 mL/min using a peristaltic pump (Tecnal, Piracicaba, SP, Brazil, model TE-BP-01)), at room temperature. The mixing rate was kept constant at 2000 rpm using a mechanical stirrer (Tecnal, Piracicaba, SP, Brazil, model TE-AM-01). For the production of membranes containing HA, the powder was added to the X solution before its combination with the C solution to reach masses of 0.4, 2, and 10 g of HA, under mechanical stirring until complete dispersion of the HA powder. The HA powder was added to the X solution because the C solution is acidic, which would cause the undesired dissolution of HA [29]. The quantities of HA added resulted in both polysaccharides to HA mass ratios (C:X:HA) of 1:1:0.4, 1:1:2, and 1:1:10, respectively. The formulations were then, correspondingly, described as C_1_X_1_HA_0.4_, C_1_X_1_HA_2_, and C_1_X_1_HA_10_. The formulation free of HA produced as a control was described as C_1_X_1_HA_0_.

The HA-polysaccharide mixture was transferred to polystyrene Petri dishes 15 cm in diameter (70 mL of suspension per dish) and dried at room temperature for 5 days. After, the membranes were immersed in 70 mL of a 3.7 g/L NaHCO_3_ solution for 24 h to neutralize the residual acetic acid from the C solution. Then, the membranes were washed with 70 mL of ultrapure water and dried in an oven (Tecnal, Piracicaba, SP, Brazil, model TE-ES-01) at 35 °C for 24 h. Finally, the membranes were sterilized by exposure to a mixture composed of 30% ethylene oxide and 70% carbon dioxide for 8 h at 40 °C, at a relative humidity between 30–80% at Acecil Central de Esterilização Comércio e Indústria Ltda. (Campinas, SP, Brazil).

### 2.3. HA and Membranes Characterization

#### 2.3.1. X-ray Diffraction (XRD)

The synthesized HA and the membranes containing HA were analyzed by XRD to identify the distinct phases. A diffractometer (Malvern Panalytical, Worcestershire, UK, model X’Pert-MPD) with an X-ray source, CuKα (λ = 0.1542 nm) emission lines from a generator operating at 40 kV and 40 mA was used. The 2θ scanning ranged from 10° to 80°, with a step size of 0.02° at 50 s/step.

#### 2.3.2. Fourier Transform Infrared Spectroscopy (FTIR)

The identification of the functional groups was performed by Fourier transform infrared spectroscopy (FTIR Prestige-21 Shimadzu, Columbia, MD, USA). The HA powder and membrane formulations were macerated together with KBr (*w*/*w*) in a ratio of 1:100 (KBr/sample). The samples were pressed in a hydraulic press (AMEF, São Paulo, SP, Brazil, model AP-25T) and analyzed in tablets. The spectra were obtained in the range of 4000 and 400 cm^−1^, with a resolution of 4 cm^−1^ and 128 scans.

#### 2.3.3. Scanning Electron Microscopy (SEM)

The analysis of the morphology of the surface of the produced membranes, as well as of the HA powder obtained, was performed using a scanning electron microscope (LEO Electron Microscopy, Cambridge, UK, model Leo 440i). The HA powder and membranes were previously coated by sputtering a layer of 200 Å of gold (EMITECH, Kent, UK, model K450).

#### 2.3.4. Thermogravimetric Analysis (TGA)

The stability of the biomaterials produced was evaluated in terms of weight variation with temperature over time by thermogravimetric analysis (TGA) and the derivative of the TGA curves (DTG) using microanalytical balance analysis (Mettler Toledo, Barueri, SP, Brazil, model MX5). The analyses were performed in alumina crucible with an initial temperature of 25 °C and final temperature of 1000 °C, using a temperature variation rate of 10 °C/minute and an N_2_ flow rate of 100 mL/minute (Mettler Toledo, Barueri, SP, Brazil, model TGA/DSC 1).

#### 2.3.5. Confocal Laser Microscopy Analysis

Laser scanning microscopy was performed in all membrane formulations after immersion in phosphate-buffered saline (PBS, product reference D5773, Sigma–Aldrich, St. Louis, MO, USA) solution and incubation for 1 h at 35 °C. Topographic images and roughness measurements were taken on both sides of the membranes by scanning the *Z*-axis in high resolution with a laser scanning confocal microscope (Keyence, Itasca, IL, USA, model VK-X200), using a wavelength of 408 nm.

#### 2.3.6. Mechanical Properties

The determination of the tensile strength and Young modulus of the produced membranes followed the ASTM D882-18 standard. The samples were cut to a size of 10 cm long and 2.54 cm wide (n = 4) and were previously hydrated with PBS solution for 1 h at 35 °C. A texturometer (Stable Micro System, Godalming, UK, model TA.XT2i) was used to perform the tests, with a cell load of 50 kgf, a gauge length of 5 cm, and a crosshead speed of 1 cm/s. The test was carried out until the rupture of the samples. The results were analyzed using Equations (1) and (2):

(1) $$T=\frac{F}{A}$$

(2) $$E=\frac{F\times l_{0}}{A\times \Delta l}\;$$

where *T* refers to the tension at break (MPa); *F* is the maximum strength (N); *A* is the cross-section area (mm^2^); *E* is the Young modulus (kPa) determined at the initial linear part of the stress versus strain curve; *l*_0_ is the initial distance between the equipment grips (m) and Δl is the distance variation at the breaking moment.

#### 2.3.7. Proliferation of DPSC on the Surface of the Membranes

All experiments in this section were performed based on adaptations of the International Organization for Standardization ISO–14644 standard using DPSC.

Before analyzing the behavior of DPSC when in contact with the membranes, the cells were characterized by immunophenotyping with the antibody markers CD105 (product reference 561443), CD73 (product reference 550257), CD90 (product reference 555596), and CD45 (product reference 347464), all from Becton Dickinson, to determine cell purity. For that, after cell detachment, 5 µL of CD105, 20 µL of CD45 and CD73 and 2 µL of CD90 antibodies were added to 1.0 × 10^5^ viable cells in test tubes containing 0.5 mL of Stain Buffer (product reference 554656, Becton Dickinson). The samples were then incubated for 30 min at 24 °C. After this period, the samples were centrifuged at 100× *g* for 5 min (Sieger, Campo Mourão, PR, Brazil, model Sirius 4000). The supernatant was discarded, and the samples were resuspended in 0.5 mL of Stain Buffer and analyzed using a flow cytometer (BD Biosciences, California, CA, USA, model BD Accuri C6) to obtain the cell marking histograms.

The proliferation of DPSC on the membranes was analyzed using the 2-(4,5-dimethyl-2-thiazolyl)-3,5-diphenyl-2H-tetrazolium (MTT) bromide salt assay (product reference M5655, Sigma-Aldrich, St. Louis, MO, USA). Dulbecco’s modified Eagle medium (DMEM, product reference D5523) supplemented with 10% (*v*/*v*) of fetal bovine serum (product reference F2561), 1% (*v*/*v*) of L-glutamine (product reference 59202C) and 1.1% (*v*/*v*) penicillin/streptomycin (product reference P4333), all from Sigma-Aldrich (St. Louis, MO, USA) were used for cell culture.

The membranes were cut into circular samples (6 mm in diameter) with a biopsy punch (Ritcher, model trepano N6) and fixed to the bottom of 96-well polystyrene plates (Corning Life Sciences) treated previously with 5.0 µL of chloroform (Sigma-Aldrich, St. Louis, MO, USA) to decrease membrane displacement in the bottom of the plate. The plates containing the membranes were then sterilized as described in Section 2.2.2. Both sides of each membrane formulation were tested (n = 3) to analyze the influence of micro-roughness resulting from the addition of HA powder. The culture medium used for cultivation was dispensed (0.2 mL) in each well and the plate was placed for 24 h at 37 °C in an incubator (Panasonic, MCO-19AIC UV) for sample hydration. After this period, the medium was discarded and replaced with the same volume of fresh medium. Then, 0.025 mL of cell suspension was added to the surface of each membrane sample to reach 2.5 × 10^4^ cells per well. Control experiments were performed by either exposing the membranes (blanks) or the cells (positive proliferative control) to supplemented culture medium.

After predetermined periods of 24, 48, and 72 h from initial cell inoculation on the membranes, aliquots of 0.02 mL of a 0.5 mg/mL MTT solution in PBS were added to the wells. The plates were incubated again at 37 °C for 4 h. At this point, the contribution of two different cell populations was assessed separately: cells adhered to the membranes, and cells adhered to the plate surfaces (described herein as neighboring cells).

For the first case, the membranes were transferred to microcentrifuge tubes and 0.2 mL of dimethyl sulfoxide (DMSO, product reference D8418, Sigma-Aldrich, St. Louis, MO, USA) was added to dissolve the formazan crystals. The samples were incubated for 30 min at 25 °C and aliquots of 0.15 mL of the solutions were then transferred to new 96-well plates for absorbance analysis at 560 nm (Promega spectrophotometer, Madison, WI, USA, model Glomax E8032).

To analyze the contribution of the cells adhered to the plate surface (neighboring cells), the MTT solution was removed and 0.2 mL of DMSO was added to dissolve the formazan crystals. After a 30 min incubation period at 25 °C, aliquots of 0.15 mL from each well were transferred to another 96-well plate to avoid interference from the bottom of the plates caused by the chloroform treatment. The results of this analysis are shown in the Supplementary Materials.

### 2.4. Statistical Analysis

Data were analyzed employing the Statistica^®^ 7 software, using one-way analyses of variance (ANOVA) with the addition of Tukey’s correction for multiple comparisons testing, with a level of significance of *p* ≤ 0.05. Results are expressed as the mean ± standard deviation.

## 3. Results and Discussion

### 3.1. Crystallinity of the Membranes

**Figure 1:**
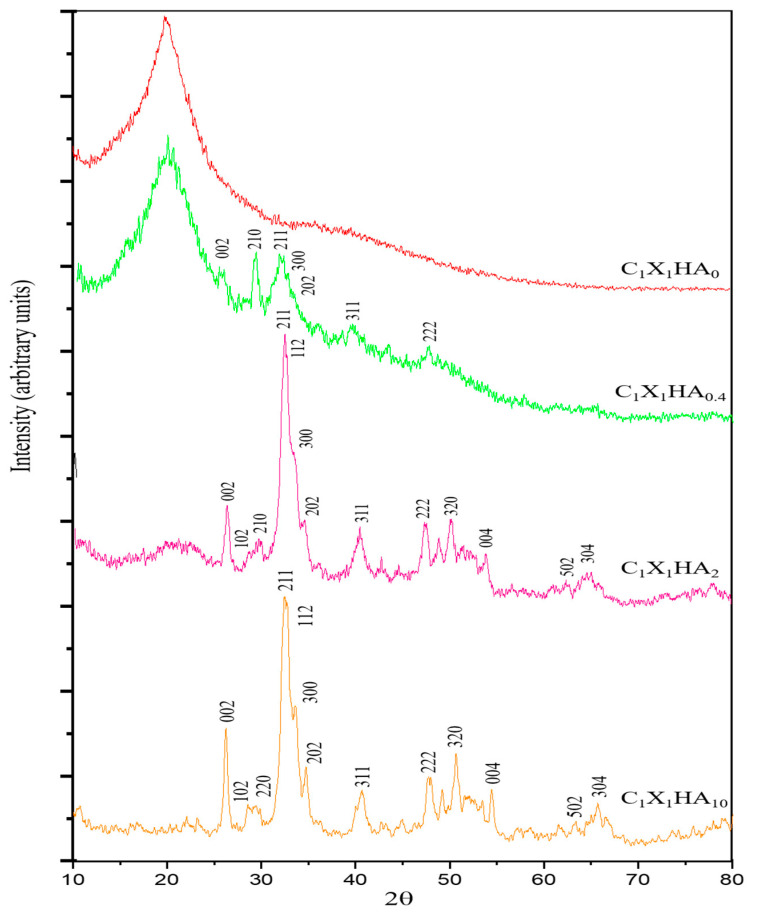
X-ray diffractograms obtained for hydroxyapatite-chitosan-xanthan gum membrane formulations and pristine HA powder.

Figure 1 shows the diffractogram patterns of the chitosan-xanthan membrane formulations produced containing or not HA.

The XRD analysis of the C_1_X_1_HA_0_ formulation shows the presence of an amorphous phase between 12° and 25°, following the results reported by Westin et al. [30] for chitosan-xanthan membranes. The increase in HA content in the C-X matrix can be associated with the presence of peaks between 25° and 35°.

The diffractograms for the C_1_X_1_HA_10_ formulation and HA powder itself (obtained by the International Center for Diffraction Data 00-009-0432, [31]) confirm that a product with low to medium crystallinity and similar to the mineral phase of human bone was obtained. The characteristic peaks of the crystalline structural plane of the HA phase, comprising the eight main peaks of angular position between 25° and 50°, were observed [32]. These results suggest that the HA powder incorporation into the C-X polyelectrolyte complex does not change the HA crystallinity and phase characteristics, despite the use of acetic acid for chitosan dissolution [29]. Neves et al. [22] found results similar to those described in this work for porous scaffolds composed of C-X and HA. In that work, the researchers also observed the crystalline phase of HA in the scaffolds. An amorphous phase related to C-X was also detected. In Figure 1, the amorphous phase between 12° to 25° decreased with the increase of HA concentration.

### 3.2. Comparative Results of Fourier Transform Infrared Spectroscopy

**Figure 2:**
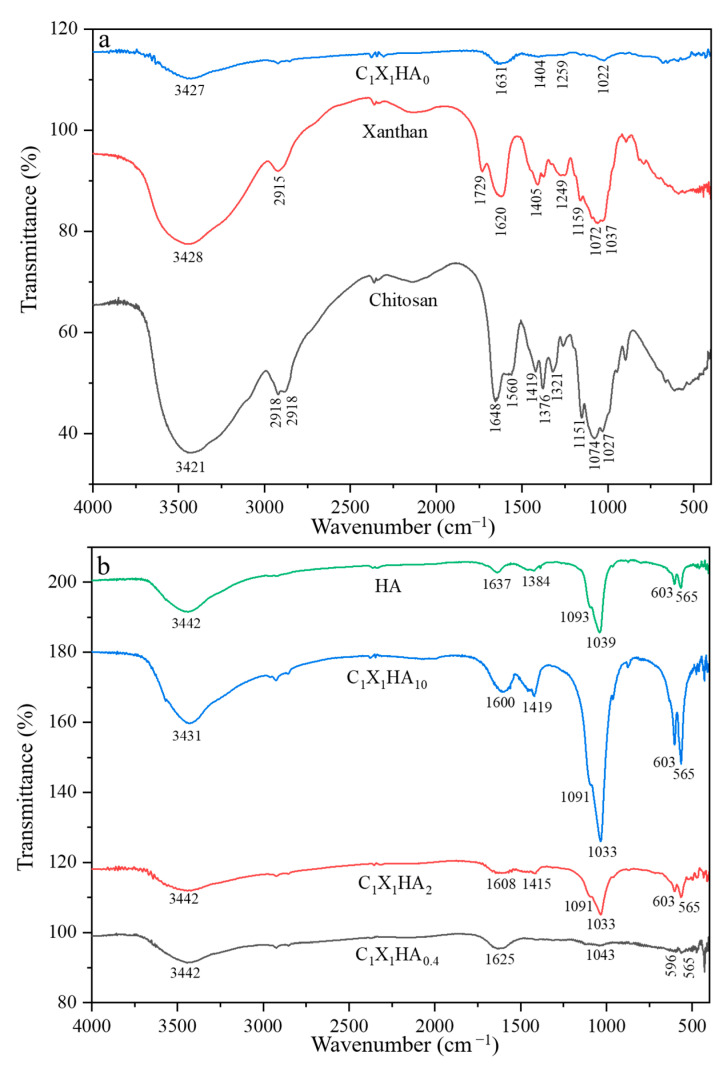
Fourier transform infrared spectra obtained for: (**a**) the chitosan-xanthan membrane without hydroxyapatite (C_1_X_1_ HA_0_) and the pristine polysaccharides chitosan and xanthan gum, and (**b**) chitosan-xanthan membrane formulations incorporating HA at different concentrations and pristine HA.

The FTIR spectra of the pristine polysaccharides chitosan and xanthan gum, of the membrane not incorporating HA, and also of the membranes containing different proportions of HA and of HA itself are shown in Figure 2.

As observed in Figure 2a, the characteristic bands of chitosan, such as the amide I groups (-C=O), -N-H_2_ (II), and -CH_2_, at 1648 cm^−1^, 1560 cm^−1^, 1376 cm^−1^, respectively, as well as the bands of xanthan gum, such as the carboxyl groups, -C=O, at 1620 cm^−1^ e 1729 cm^−1^, from acetate and pyruvate, respectively, were observed. Besides, the band at 3430 cm^−1^, attributed to the stretching vibrations of hydroxyl, and the bands between 2879 cm^−1^ and 2918 cm^−1^, related to the symmetrical and asymmetrical vibrations of -C-H, are also present. The absorption of bands between 800 cm^−1^ to 1200 cm^−1^ correspond to the glycosidic bonds of the rings present in both pristine polysaccharides, such as -C-O-C; -C-O, and -C-C [33,34].

The FTIR spectrum of the C_1_X_1_HA_0_ membrane indicated the polyelectrolyte complex formation between both C and X polymers due to the intensity of transmittance, band displacement, and the low absorption (high transmittance value) of some groups, as shown in Figure 2a. The absorption intensity related to the stretching of O-H groups, as well as the narrowing of its band, indicates hydrogen bond formation between these polymers [35,36]. In addition, it is possible to observe the formation of a band with low absorption at 1022 cm^−1^, related to the glycosidic bonds present in C and X chains [35]. Similar results were reported by Popa et al. [37] and Ćirić et al. [38], however with a higher vibration than that found in the present work.

The low absorption intensity of the C_1_X_1_HA_0_ formulation indicates that the groups are not free to absorb the IR radiation, suggesting a medium-to-strong interaction of the functional groups [38]. The presence of a single peak at 1631 cm^−1^, resulting from the displacement of the 1648 cm^−1^ peak from chitosan, as well as the disappearance of the amide groups at 1560 cm^−1^ and 1736 cm^−1^ for X and C, respectively, are observed. This region (1560 to 1736 cm^−1^) can be related to the vibration of the carboxyl and amino groups, which suggests an interaction between them and, consequently, the polyelectrolyte complex formation [35,36].

As shown in Figure 2b, the intensity of the bands between 560 cm^−1^ and 1095 cm^−1^, related to the vibration of -PO_4_^−3^ groups, increased when the HA concentration in the membranes was raised, similar to data previously reported in the literature [24,25,26]. These results suggest that with the increase in the vibration of the bands related to -PO_4_^−3^, interactions within the polysaccharide matrix via hydrogen bonds or ionic interactions with Ca^2+^ could have occurred [27,28]. In these spectra, the region between 3400 cm^−1^ and 3500 cm^−1^ corresponds to water sorption by HA and polysaccharides [22,39]. Close bands between 1030 and 1090 cm^−1^ were also reported for the production of scaffolds for bone healing composed of chitosan and hydroxyapatite [39]. The bands in this region were related to the saccharide C-O-C stretching of chitosan and the phosphate of HA [39].

### 3.3. Thermal Stability of HA and Membrane Formulations

**Figure 3:**
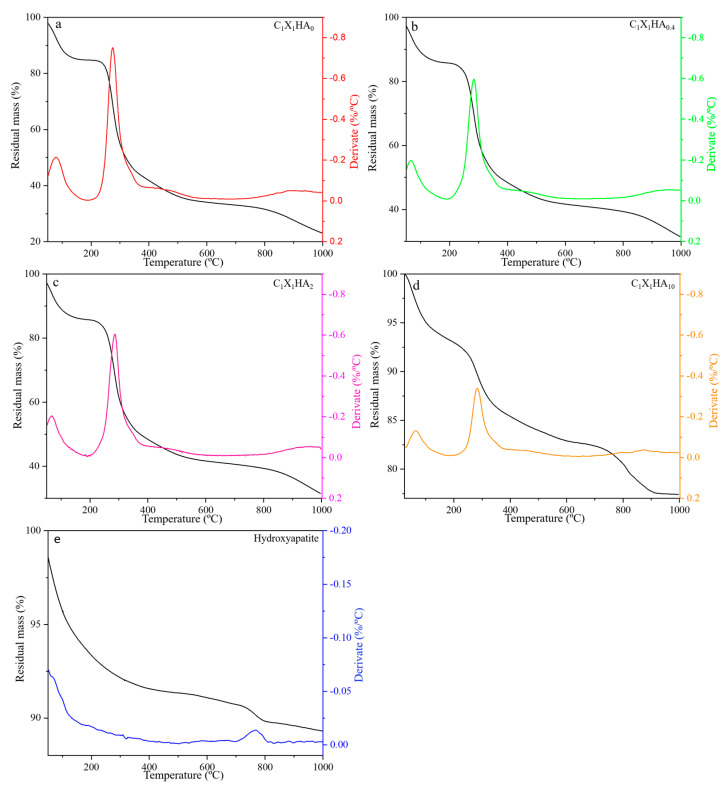
Thermogravimetric analysis and its derivative for the membrane formulations: (**a**) C_1_X_1_HA_0_; (**b**) C_1_X_1_HA_0.4_; (**c**) C_1_X_1_HA_2_; (**d**) C_1_X_1_HA_10_; and (**e**) pristine hydroxyapatite.

The influence of the presence of HA on the thermal behavior of different membrane formulations was evaluated by thermogravimetry. The formulations C_1_X_1_HA_0_ (Figure 3a), C_1_X_1_HA_0.4_ (Figure 3b), and C_1_X_1_HA_2_ (Figure 3c) presented similar degradation rates, exhibiting mass loss in three stages. The first thermal event occurs between 30 °C and 120 °C at a relatively low rate. Westin et al. [30,39] attributed this event to water loss, which in the present study represents a variation of around 13% of mass loss. The second thermal event occurred between 250 °C and 350 °C, approximately, with a maximum degradation rate at 300 °C, according to the DTG. The mass loss in the second stage is approximately 60% for the C_1_X_1_HA_0_ formulation and 45% for the C_1_X_1_HA_0.4_ and C_1_X_1_HA_2_ formulations, which can be attributed to depolymerization of chitosan and degradation of the xanthan gum chains [30,40]. The residual mass at the third stage ranges from 25% for C_1_X_1_HA_0_ to 35% for C_1_X_1_HA_0.4_ and C_1_X_1_HA_2_ formulations, as a simultaneous result of both the increased HA incorporation and possible HA dihydroxylation and/or decarbonation of its CO_3_^2-^ groups [41].

The C_1_X_1_HA_10_ formulation, containing the highest proportion of HA (83% *w*/*w*), showed a different thermal behavior when compared to the other formulations (Figure 3d). Higher HA concentration led to a decrease in thermal degradation, resulting in a residual mass of 77%. This behavior can be associated with the presence of hydroxyl, carboxyl, and amino groups in the polymer chains, which promote the occurrence of hydrogen bonds and interactions between the surface of the HA particles and the polymers [42].

As a consequence, an increase in the decomposition temperature of the C_1_X_1_HA_10_ formulation was observed [43,44]. The DTG at 810 °C indicates a slight mass loss, probably related to the decomposition of HA into CaO [42]. The mass loss related to this decomposition is also observed in Figure 3e for the HA powder, however, at a lower temperature, around 700 °C, which corroborates the aforementioned statement about the increased decomposition of the material related to the interaction between the polysaccharides and HA particles. Unlike the other formulations, mass loss was not observed for the C_1_X_1_HA_10_ membrane above 900 °C. Except for these three events, the DTG curves behave fairly constantly above 400 °C, with a reasonably steady rate of mass loss.

### 3.4. Morphology Surface Analysis of HA Powder and Membranes

**Figure 4:**
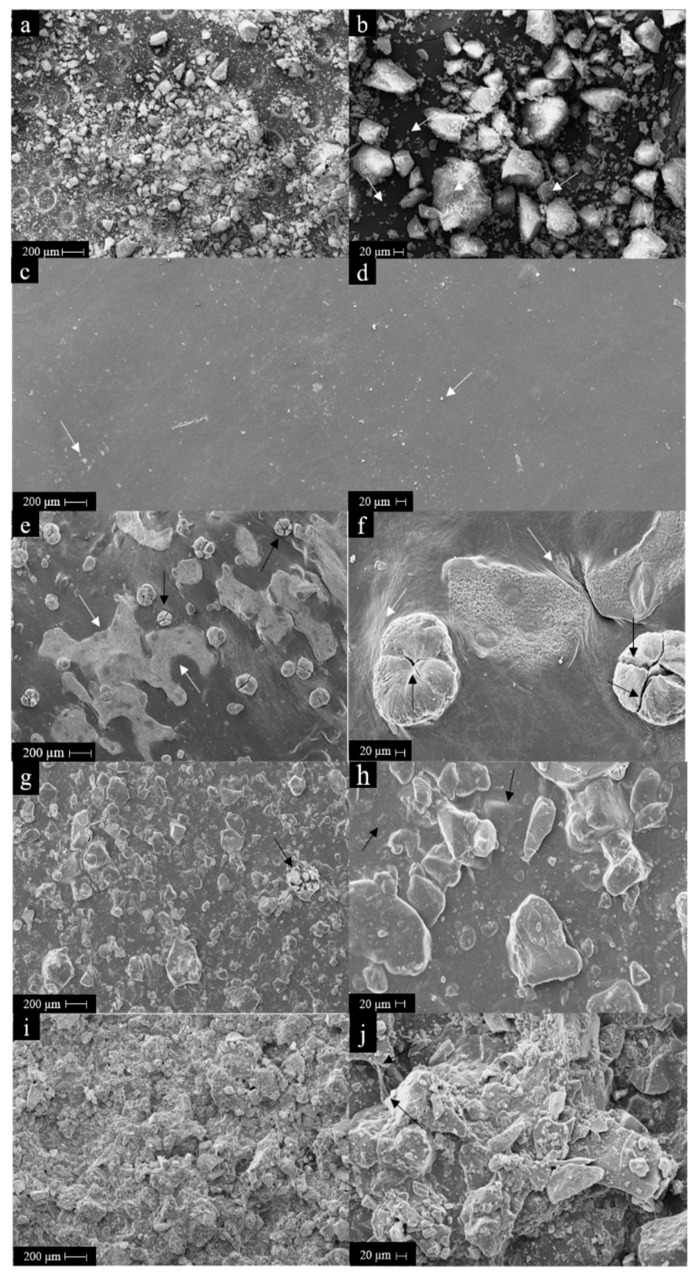
SEM of HA powder and membranes surface at different magnifications. (**a**,**b**) Aspect of the HA powder obtained in the synthesis process. Aspect of the surface of the different membrane formulations: (**c**,**d**) C_1_X_1_HA_0_; (**e**,**f**) C_1_X_1_HA_0.4_; (**g**,**h**) C_1_X_1_HA_2_; (**i**,**j**) C_1_X_1_HA_10_. The black and white arrows highlight particular characteristics observed during the analysis of the results.

The results on the morphology of HA powders and the membranes analyzed by SEM can be observed in Figure 4.

HA particle aggregates with irregular morphology were obtained (Figure 4a,b), with sizes up to 150 µm, as expected after sieving. A large number of smaller particle aggregates, with sizes equal to or less than 20 µm, were also observed (as indicated by the white arrows). With the increase of surface area to volume ratio for the HA nanometric particles, as observed in Figure 4b, electrostatic forces and surface tension became dominant, increasing particle–particle interactions which then induced aggregation, as also observed by Ma and Lim [45].

As shown in Figure 4c,d, the C_1_X_1_HA_0_ formulation showed smooth and uniform surfaces, as also evidenced by Bombaldi de Souza et al. [34] for chitosan-xanthan gum membranes. The small fragments observed on the membrane surface by SEM analysis, indicated by the white arrows, may be residual particles of sodium bicarbonate used during membrane neutralization.

The micrographs of the membrane formulations containing HA powders, shown in Figure 4e–j, indicate an increase in roughness with increasing HA content. In the C_1_X_1_HA_2_ (Figure 4g,h) and C_1_X_1_HA_10_ (Figure 4i,j) formulations, similar HA aggregates as described for C_1_X_1_HA_0.4_ membranes can be observed.

Nevertheless, in the C_1_X_1_HA_0.4_ formulation (Figure 4e,f), aggregates of either small or larger particles can be seen on the surface. These smaller particles are concentrated in large areas of the membrane surface, as indicated by the white arrows (Figure 4e). The larger blocks observed on the surface of this formulation (as the one pointed out by the black arrow) are formed by two or more units of particle aggregates, which are covered by a thin layer of the membrane. However, there is a central region of these large blocks that are not covered by the polymer layer. This region appears outlined by cracks and is indicated by the black arrows (Figure 4e,f).

Several factors may be related to the agglomeration of these particles, such as the charge of the functional groups, the hydrophilicity, molecular weight and flexibility of the polymer chains, and free interfacial energy of HA, chitosan, and xanthan gum in the mixture [45,46]. For all formulations, the selective agglomeration of HA particles on one side of the surface of membranes probably occurred during the drying step and polymer matrices crosslinking.

In the C_1_X_1_HA_2_ and C_1_X_1_HA_10_ formulations, particles were also noticed at random distribution, in different sizes, aggregated or not, as shown in Figure 4g,j. For the C_1_X_1_HA_2_ formulation (Figure 4g), it was possible to observe a similar structure to that of C_1_X_1_HA_0.4_, containing large HA blocks (indicated by the black arrow). As shown in Figure 4h, some of the HA aggregates are found buried within the polymeric matrix (pointed out by black arrows). In the C_1_X_1_HA_10_ formulation, small particles and large aggregates overlap due to the high HA concentration (Figure 4i,j). The evidence of the interaction of the polysaccharide matrix surface with the HA particles is observed in Figure 4j, in which the fiber formed by the C-X polyelectrolyte complex adheres to the surface of an HA block (indicated by black arrows), as mentioned in Section 3.2.

### 3.5. Membrane Aspect Evaluated by Confocal Microscopy and Roughness

When observed with the naked eye, the membrane side that was in contact with the Petri dish surface during membrane drying (Side 1) was shown to be smoother than the side exposed to the atmosphere (Side 2). Due to high water absorption by the polysaccharides [47], the membranes significantly increased their dimensions, and potential changes might be also expected in membrane topography in these conditions. Since membrane aspect and texture are analyzed solely by visual inspection and SEM may not provide sufficient information to infer membrane behavior in wet conditions, and given that confocal microscopy allows the analysis of wet samples, the use of this technique may favor results obtained in conditions similar to those of clinical practice.

**Figure 5:**
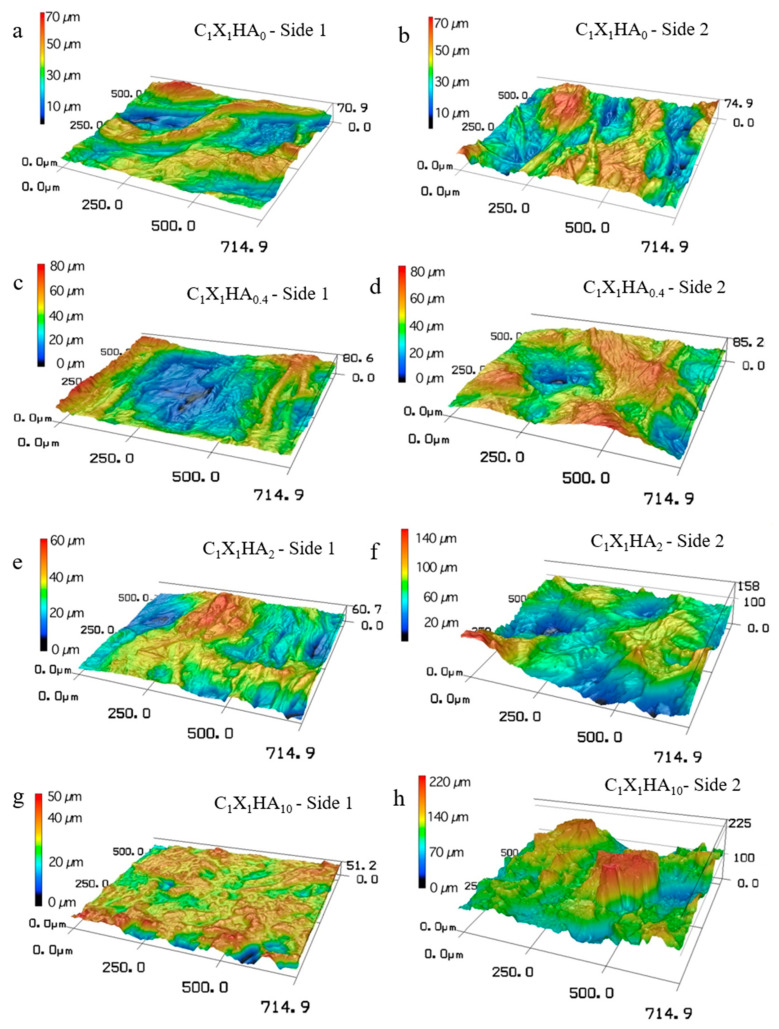
Three-dimensional images of membranes exposed to PBS solution at 35 °C for 1 h obtained by confocal microscopy. Side 1 corresponds to the surface that was in contact with the Petri dish during the drying step of the membrane fabrication (smoother surface), while side 2 refers to the surface exposed to the atmosphere (rough surface): (**a**,**b**) side 1 and 2 for C_1_X_1_HA_0_; (**c**,**d**) side 1 and 2 for C_1_X_1_HA_0.4_; (**e**,**f**) side 1 and 2 for C_1_ X_1_HA_2_; (**g**,**h**) side 1 and 2 for C_1_X_1_HA_10_.

Therefore, visual and SEM observations were confronted with topography analysis by confocal microscopy of membranes previously exposed to PBS solution for 1 h (results shown in Figure 5). The corresponding root means square roughness (Rq) values of both sides of the membranes are indicated in Table 1.

The analysis of the roughness on the side 2 surfaces showed that the increase in the HA concentration from C_1_X_1_HA_0_ to C_1_X_1_HA_2_ also led to an increase in Rq. However, there was no significant difference between the C_1_X_1_HA_2_ and C_1_X_1_HA_10_ formulations. On the other hand, a decreased tendency was observed for surface roughness on side 1 (smoother) with the increase of HA content, considering the average values.

The roughness of the side 1 surface of the C_1_X_1_HA_0.4_ and C_1_X_1_HA_2_ formulations is comparable to that of C_1_X_1_HA_0_. In addition, the comparison between both sides of the same formulation showed that Rq is significantly different only for the C_1_X_1_HA_2_ and C_1_X_1_HA_10_ formulations, which presented the highest roughness on side 2 (rougher). Similar results were obtained by Bombaldi de Souza et al. [48], with an Rq value of 6.24 ± 1.39 µm for dry C-X membranes.

The literature has shown an improvement in cellular behavior with a combination of nano and micrometric surface roughness features [47,49,50]. The occurrence of microroughness on a surface (on micron and submicron scales) mainly affects cell adhesion and growth, while nanometric scale roughness may regulate the subcellular sensing mechanism. From a biological point of view, fibroblasts, typical of connective tissue, attach and grow better on smooth surfaces than on micro-rough surfaces [49]. On the contrary, osteoblastic cells may attach and grow more rapidly on rough surfaces, favoring the osteoconduction process [50]. Therefore, the design of different topographies on both sides of the membranes may be relevant when aiming at a smooth surface in direct contact with the soft tissue and a rough surface to guide the bone regeneration process on the defect side.

### 3.6. Mechanical Properties

Considering the application in guided bone regeneration, the biomaterial must have appropriate mechanical resistance to support the underlying tissues and resist exposure to the environment of the oral cavity without suffering local disruption [51]. In the present work, the study of the mechanical properties of the biomaterials was performed to evaluate how the incorporation of different HA concentrations in the membranes would influence these properties. As when in vivo the membranes would remain in direct contact with biological fluids, the samples were previously soaked in PBS for 1 h to simulate the in vivo environment. The results achieved are shown in Table 2.

The membrane formulation containing HA with the highest mechanical tensile strength was C_1_X_1_HA_2_, possibly as a result of improved interaction between the ceramic phase and the polysaccharides, which is related to the polysaccharides functional groups and the free interfacial energy of HA [45,46]. However, as the mass of HA increases to 10 g, the HA to polymer ratio increases significantly, reducing the number of interactions between the polysaccharide molecules themselves and of these polymers with HA molecules, affecting the cohesion of the final matrix.

Westin et al. [50] and Bellini et al. [51] also produced chitosan-xanthan membranes and reported higher values of tension at the break, respectively 1.33 ± 0.26 and 12.7 ± 2.0 MPa, in comparison to the C_1_X_1_HA_0_ formulation in the present work. However, these authors performed their analyses on dry samples, while in this work, all the analyses were performed after the immersion of the membranes in PBS to simulate the conditions of use. Bombaldi de Souza et al. [52] reported decreasing tension at the break, ranging from 9.4 to 0.9 MPa, when C-X membranes were exposed to increasing relative humidity, from 22% to 100%, respectively. The maximum value reported by the last authors is still higher than those obtained herein for C_1_X_1_HA_0_ (around 0.29 MPa) because of the higher mass ratio of polysaccharides per cubic volume used by Bombaldi de Souza et al. [52].

The tensile strength of films containing chitosan/polyvinyl alcohol incorporating or not HA nanoparticles was analyzed by Prakash et al. [53]. A slight increase in tensile strength, from 33.8 ± 0.5 to 35.2 ± 0.5 MPa, was observed when the ceramic compound was added to the formulation. This result was also attributed to the hydrogen bonds formed between the polymers and the HA particles added to the film.

The C_1_X_1_HA_0.4_ formulation presents the highest Young modulus in comparison to all formulations containing HA tested in this work. A similar trend was observed by Salim et al. [54] in the production of scaffolds composed of polyvinyl alcohol-hyaluronan (PVA/HAc) incorporating HA. For the formulation containing 1% in mass of HA nanoparticles, values close to 218 MPa were obtained, while for the formulation without HA, the Young modulus was around 35% lower. On the other hand, in this work, with the increase of the mass of HA particles of up to 150 µm, the Young modulus decreased, reinforcing the ideal HA-polymer ratio mentioned before.

### 3.7. In Vitro Cell Proliferation on Both Surfaces of Membranes

Adhesion and cell growth on a biomaterial surface can be affected by several factors, such as surface composition, charge, and bioactivity, as well as its hydrophilic/hydrophobic balance, roughness, and stiffness [55]. As confirmed by confocal microscopy and mechanical properties, the membranes produced have sides with different roughness and topography, therefore, both sides were analyzed. The behavior of mesenchymal stem cells on both membrane surfaces was analyzed in terms of cell proliferation from 24 to 72 h, and the results are shown in Table 3.

Data regarding the characterization of the cells used throughout this work are available in the Supplementary Materials, in Figures S1–S3 and Table S1. The cells showed adhesion capacity, fibroblast-like cell morphology, positive immunophenotypic markers for mesenchymal stem cells (CD105, CD90, and CD73) above 96%, and negative immunophenotypic markers (CD45) below 1%, in accordance with the established criteria for DPSC, showing therefore to be adequate for the proposed study [56]. The results of the absorbance of the positive controls of the MTT assay (shown in Table S2 of the Supplementary Materials) show a behavior trend analogous to that observed for the cells in culture shown in Figure S3 of the Supplementary Materials.

When comparing the results of the proliferation assay with the roughness data presented in Table 1, the C_1_X_1_HA_10_ formulation also showed the lowest roughness among the HA-containing formulations when analyzing membranes side 1. In addition to the roughness aspect, the stiffness of the materials can influence cell adhesion and, consequently, cell proliferation. Generally, less rigid materials lead to weaker cell attachment, and most cells, including mesenchymal stem cells, are dependent on adhesion for multiplication [57,58]. Another phenomenon to consider is durotaxis, in which stem cells move from a soft tissue toward a more rigid tissue [58,59]. The incorporation of powdered HA into the membranes increases the material’s stiffness, favoring cell adhesion and proliferation, as observed for the C_1_X_1_HA_10_ formulation. On the other hand, the results of the C_1_X_1_HA_0_ formulation suggest good initial cell adhesion and proliferation at 48 h and, probably, after this period, the cells may have migrated to the surface of the polystyrene cell culture plate, due to durotaxis [57,59], as indicated in Tables S3 and S4 of the Supplementary Materials. Differently from the smooth surface results, the C_1_X_1_HA_10_ formulation showed superior results when compared to all other formulations at all time points tested for the membrane side with a rough surface (Table 3). As observed by SEM (Figure 4i,j), HA strongly agglomerates on the C_1_X_1_HA_10_ surface, increasing the number of focal adhesion points, and this can be a factor that stimulates subsequent mesenchymal stem cells proliferation (Supplementary Materials) [55,57,58].

At 24 and 48 h, except for the C_1_X_1_HA_10_ formulation, similar values of percentage of proliferation were exhibited by cells on the rougher surfaces of all other matrices (Table 3). The same behavior can be observed in the kinetic growth curves of stem cells from the third molar dental pulp, for which the start of the exponential growth phase may occur after 48 h, after the lag phase [58], despite the DPSC used herein showed a shorter lag phase when cultured on polystyrene surfaces (as shown in Figure S3 of the Supplementary Materials). Therefore, considering the inoculation of the DPSC on biomaterials with different surface roughness and rigidity, the duration of both the lag phase and exponential cell growth may vary.

Since C_1_X_1_HA_10_ membranes show a higher roughness value on side 2, but lower on side 1, with results statistically equivalent to those of the C_1_X_1_HA_2_ formulation, this property alone cannot explain the behavior observed. The addition of HA as a bioactive compound may have contributed to the proliferation of DPSCs on the polymeric matrices and its bioactivity may also have potentially accelerated different biological responses.

This behavior can also be observed when analyzing the performance of neighboring cells adhered to the surface of the plate, shown in the Supplementary Materials (Tables S3 and S4). With the increase of HA concentration in the formulations, the cells seem to prefer the membrane surface, with low migration to the bottom of the plate.

Additional analyses can be performed to further explore important characteristics related to biomaterials used in regeneration, such as wettability and the uptake of physiological fluids. The in vitro protocol described in this work also showed successful adhesion and proliferation of DPSC on the material. Due to the immunomodulatory potential of DPSC, cellularized biomaterials are shown to be excellent tools for tissue regeneration of lesions caused by periodontitis [1].

## 4. Conclusions

The synthesis of hydroxyapatite by precipitation in an aqueous medium produced a compound suitable for bone regeneration, given its similarity with the compound found in native bone tissues. Its combination with the mucoadherent polysaccharides chitosan and xanthan, in the form of dense composite membranes, was successfully achieved and no alterations in HA crystallinity and phase behavior were observed. The membranes were show to be mechanically resistant and thermally stable under physiological conditions.

Given that the selective agglomeration of HA particles was observed on one side of the membranes, biomaterials with different topographies and adequate for cell adhesion and proliferation could be produced. While the smooth surface could be placed in direct contact with the soft tissue, the rougher area could be used to guide the bone regeneration process on the defect site.

The study of the incorporation of different proportions of HA in chitosan-xanthan membranes indicated that the appropriate balance between biological and mechanical properties was obtained for the C_1_X_1_HA_2._ Further studies should be performed to determine if there is a more adequate C:X:HA mass proportion between formulations C_1_X_1_HA_0.4_ and C_1_X_1_HA_10_ for specific applications in bone tissue regeneration and also the potential of the use of these biomaterials as drug carriers, e.g., of doxycycline, which is one of the main drugs used in the treatment of periodontitis.

## Figures and Tables

**Table 1:** Surface roughness (Rq) of C-X membranes added or not with HA determined by confocal microscopy analysis.

Formulations	Root Mean Square Roughness (µm)	
	Side 1	Side 2
C_1_X_1_HA_0_	9.97 ± 1.82 ^a,A^	10.23 ± 3.21 ^a,A^
C_1_X_1_HA_0.4_	9.98 ± 1.25 ^a,A^	11.15 ± 2.22 ^a,A^
C_1_X_1_HA_2_	8.03 ± 4.98 ^a,A,B^	21.30 ± 2.56 ^b,B^
C_1_X_1_HA_10_	4.51 ± 1.05 ^a,B^	27.78 ± 12.57 ^b,B^

Different lowercase letters in the same line and different capital letters in the same column indicate a significant difference between the mean values at 95% confidence limits (Tukey test).

**Table 2:** Tensile strength and Young modulus of chitosan-xanthan membranes prepared with different concentrations of hydroxyapatite after exposure to PBS for 1 h.

Formulation	Tension at Break (kPa)	Young’s Modulus (kPa)
C_1_X_1_HA_0_	288.17 ± 76.55 ^a^	5.27 ± 0.02 ^a^
C_1_X_1_HA_0.4_	50.51 ± 11.17 ^b^	7.29 ± 0.23 ^b^
C_1_X_1_HA_2_	211.59 ± 16.12 ^c^	5.35 ± 0.01 ^a^
C_1_X_1_HA_10_	83.28 ± 9.64 ^b^	3.43 ± 0.02 ^c^

Different letters in the same column indicate a significant difference between the mean values (Tukey’s test, *p* < 0.05).

**Table 3:** Percentage values of cell proliferation over the smoother and rougher surfaces (sides 1 and 2, respectively) of the membranes in comparison to the control (polystyrene culture plate).

**Time** **(h)**	**Cell Proliferation on Side 1 (%)**			
	**C_1_X_1_HA_0_**	**C_1_X_1_HA_.4_**	**C_1_X_1_HA_2_**	**C_1_X_1_HA_10_**
**24**	41.5 ± 3.7 ^a,A^	39.4 ± 4.6 ^a,b,A^	39.9 ± 2.0 ^a,b,A^	32.3 ± 1.7 ^b,A^
**48**	56.2 ± 2.0 ^a,B^	40.5 ± 4.0 ^b,A^	39.0 ± 2.6 ^b,A^	41.6 ± 5.5 ^b,A^
**72**	40.9 ± 1.0 ^a,A^	43.5 ± 2.1 ^a,A^	49.6 ± 8.6 ^a,A^	76.9 ± 12.9 ^b,B^
	**Cell proliferation on Side 2 (%)**			
	**C_1_X_1_HA_0_**	**C_1_X_1_HA_0.4_**	**C_1_X_1_HA_2_**	**C_1_X_1_HA_10_**
**24**	41.5 ± 1.4 ^a,A,B^	35.9 ± 2.5 ^a,A^	37.2 ± 3.1 ^a,A^	53.1 ± 3.3 ^b,A^
**48**	36.0 ± 4.3 ^a,A^	35.2 ± 4.0 ^a,A^	37.1 ± 3.4 ^a,A^	55.1 ± 3.5 ^b,A^
**72**	50.8 ± 7.1 ^a,B^	66.0 ± 8.9 ^a,B^	59.5 ± 5.2 ^a,B^	91.6 ± 7.3 ^b,B^

Different lowercase letters in the same line and different capital letters in the same column for the same type of membrane surface indicate a significant difference between the mean values (Tukey’s test, *p* < 0.05). Percentage values consider optic density at 560 nm between 0.0–1.0 range.

## Data Availability

Data available on request.

## Supplementary Materials

The following supporting information can be downloaded at: https://www.mdpi.com/article/10.3390/pharmaceutics15020705/s1, Figure S1—Typical morphology of the DPSC cell line used in the in vitro assays observed by optical microscopy (10X objective); Figure S2—Histograms obtained by immunophenotyping the DPSC analyzed by flow cytometry; Figure S3—Growth curve of the dental pulp mesenchymal cells obtained in in vitro culture using 6-well polystyrene plates, expressed in terms of the variation of viable cell concentration with time. The culture medium was changed every 72 h; Table S1—Values obtained from immunophenotyping markers by flow cytometry for the cell population in the 8th passage (BD—model: Accuri C6); Table S2—Absorbance of positive controls for MTT tests; Table S3—Percentage of cells which proliferate on the surface of the culture plate, in the neighborhood of membranes exposing Face 1, when compared to control cell culture experiments; Table S4—Percentage of cells which proliferate on the surface of the culture plate in the neighborhood of membranes exposing Side 2, when compared to control cell culture experiments.
