# Supplementary material for: Cell-Friendly Chitosan-Xanthan Gum Membranes Incorporating Hydroxyapatite Designed for Periodontal Tissue Regeneration
Source: Pharmaceutics. 2023 Feb 20;15(2):705. doi: 10.3390/pharmaceutics15020705 (PMC9962096; doi:10.3390/pharmaceutics15020705)
Supplement: Supplementary file 1 [file pharmaceutics-15-00705-s001.zip › pharmaceutics-2140171-supplementary.pdf]

SUPPLEMENTARY MATERIAL

Cell-friendly chitosan-xanthan gum membranes incorporating hydroxyapatite designed for periodontal tissue regeneration

Rafael Maza Barbosa[1,2], Daniel Navarro da Rocha[2], Renata Fracielle Bombaldi de Souza[1], Jheison L. Santos[3], José Ricardo M. Ferreira[2], Ângela M. Moraes [1,*]

[1] Department of Engineering of Materials and of Bioprocesses, School of Chemical Engineering, University of Campinas, Campinas 13083-852, SP, Brazil

[2] Department of Bioengineering, R-Crio Stem Cells, Campinas 13098-324, SP, Brazil

[3] Department of Physics, Federal Rural University of Rio de Janeiro, Rio de Janeiro 23890-000, RJ, Brazil

* Correspondence: ammoraes@unicamp.br; Tel.: +55-19-3521-3921

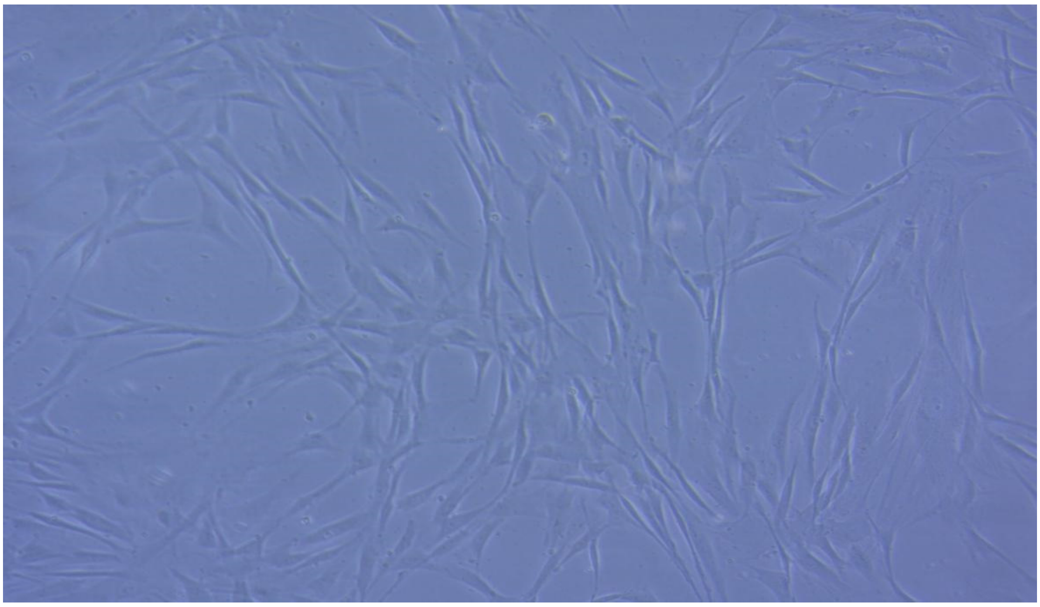

Figure S1 – Typical morphology of the DPSC cell line used in the *in vitro* assays observed by optical microscopy (10X objective).

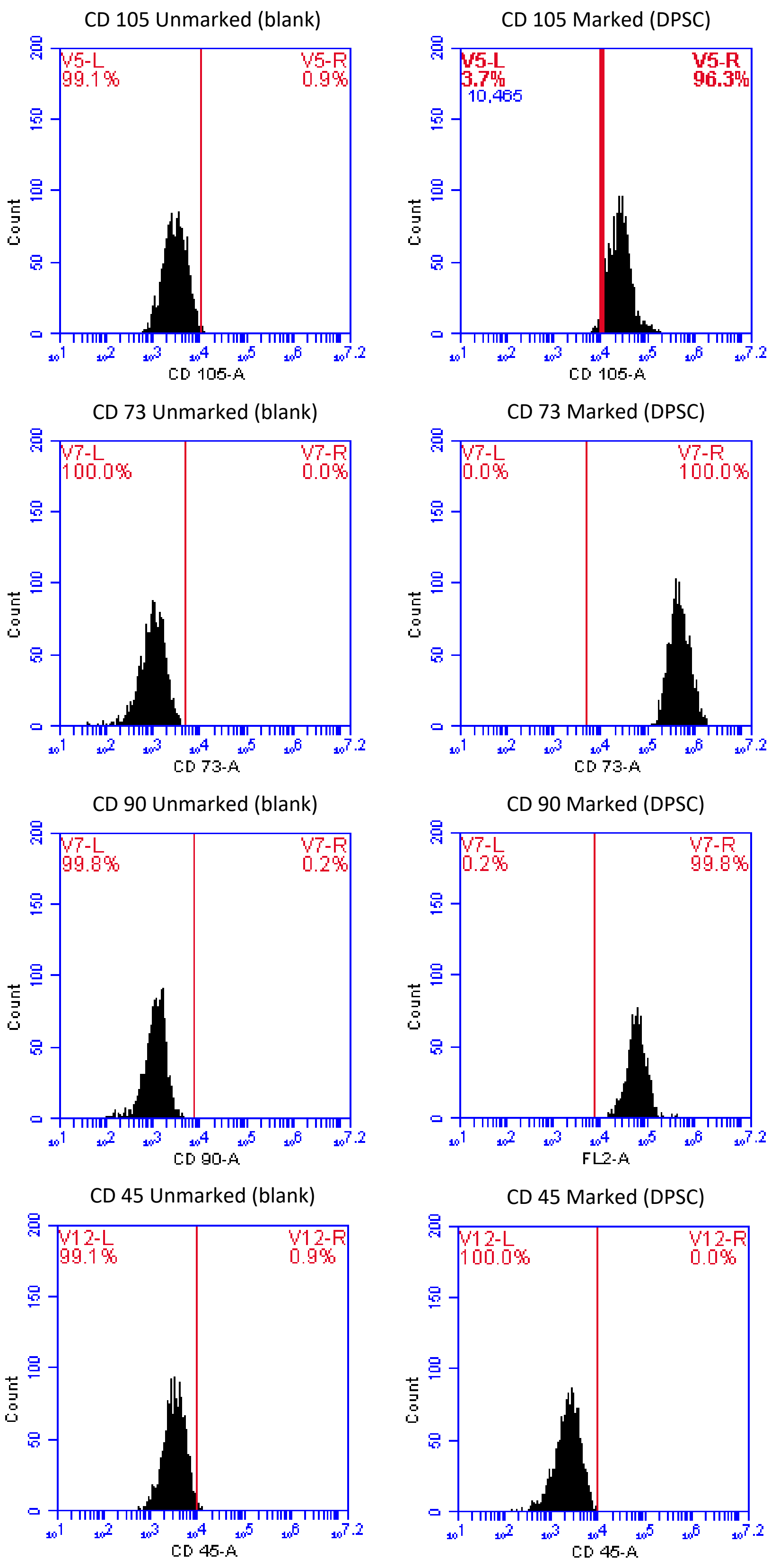

Figure S2 – Histograms obtained by immunophenotyping the DPSC analyzed by flow cytometry.

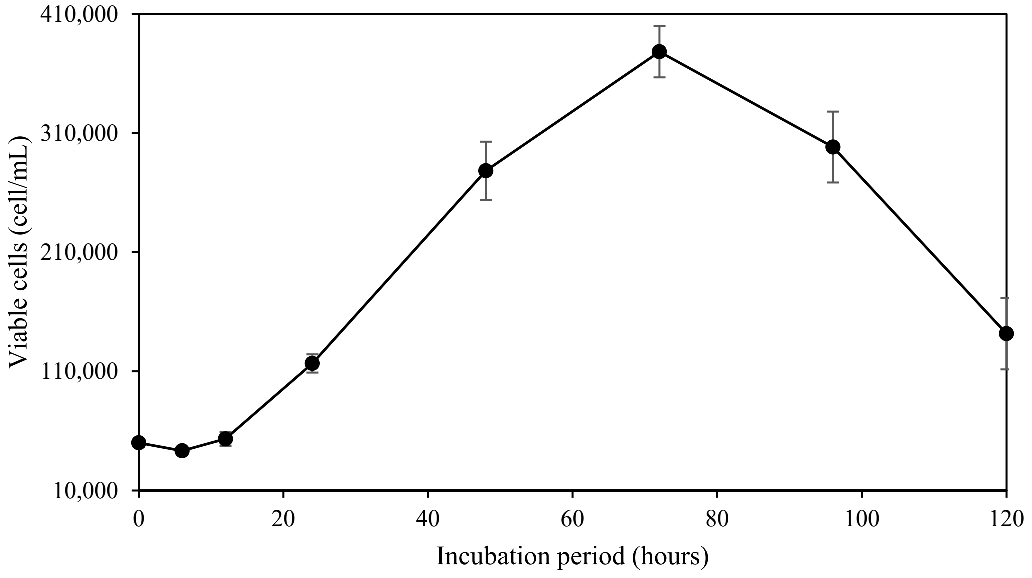

Figure S3 – Growth curve of the dental pulp mesenchymal cells obtained in *in vitro* culture using 6-well polystyrene plates, expressed in terms of the variation of viable cell concentration with time. The culture medium was changed every 72 h.

Table S1 – Values obtained from immunophenotyping markers by flow cytometry for the cell population in the 8th passage (BD – model: Accuri C6).

Immunophenotyping	Markers			
	**CD 105**	**CD 90**	**CD 73**	**CD 45**
**Expression (%)**	96.3	99.8	100.0	0.9

Table S2 – Absorbance of positive controls for MTT tests.

Time (h)	Absorbance at 560 nm
**24**	0.683 ± 0.06
**48**	1.017 ± 0.02
**72**	0.8517 ± 0.01

Table S3 – Percentage of cells which proliferate on the surface of the culture plate, in the neighborhood of membranes exposing Side 1, when compared to control cell culture experiments.

Time (h)	Cell proliferation on the neighborhood of membranes – Side 1 (%)			
	**$C_1X_1HA_0$**	**$C_1X_1HA_{0.4}$**	**$C_1X_1HA_2$**	**$C_1X_1HA_{10}$**
**24**	$62.44 \pm 5.21^{a,A}$	$42.16 \pm 8.11^{b,A}$	$28.23 \pm 3.36^{c,A}$	$13.99 \pm 1.19^{d,A}$
**48**	$22.33 \pm 5.59^{a,B}$	$42.16 \pm 5.31^{b,A}$	$39.69 \pm 2.20^{b,A}$	$18.09 \pm 7.94^{a,A}$
**72**	$53.36 \pm 11.30^{a,A}$	$74.46 \pm 2.93^{a,B}$	$55.30 \pm 7.90^{a,B}$	$20.55 \pm 9.75^{b,A}$

Different lowercase letters in the same line and different capital letters in the same column indicate a significant difference between the mean values (Tukey's test, $p < 0.05$). *Percentage values consider optic density at 560 nm between the 0.000 - 1.000 range.

Table S4 – Percentage of cells which proliferate on the surface of the culture plate in the neighborhood of membranes exposing Side 2, when compared to control cell culture experiments.

Time (h)	Cell proliferation on the neighborhood of membranes – Side 2 (%)			
	**$C_1X_1HA_0$**	**$C_1X_1HA_{0.4}$**	**$C_1X_1HA_2$**	**$C_1X_1HA_{10}$**
**24**	$58.68 \pm 10.39^{a,A}$	$48.21 \pm 7.38^{a,b,A}$	$33.38 \pm 6.41^{b,A}$	$10.22 \pm 2.60^{c,A}$
**48**	$38.99 \pm 15.29^{a,A}$	$46.51 \pm 5.99^{a,A}$	$36.86 \pm 4.76^{a,b,A}$	$15.51 \pm 1.24^{b,A}$
**72**	$53.48 \pm 11.99^{a,A}$	$61.03 \pm 9.16^{a,A}$	$43.30 \pm 10.86^{a,A}$	$17.04 \pm 5.33^{b,A}$

Different lowercase letters in the same line and different capital letters in the same column indicate a significant difference between the mean values (Tukey's test, p <0.05). *Percentage values consider optic density at 560 nm between the 0.000 - 1.000 range.
